# Uninvited guests: diversity and specificity of *Trypanosoma* infections in frog-biting midges (*Corethrella* spp.)

**DOI:** 10.1186/s13071-025-06993-8

**Published:** 2025-08-15

**Authors:** Maximilian Sigl, Jonas Virgo, Ulmar Grafe, Jonas Henske, Maximilian Schweinsberg, Thomas Eltz

**Affiliations:** 1 Department of Animal Ecology, Evolution and Biodiversity, Ruhr‐University Bochum, Bochum, Germany; 2 Faculty of Science, University Brunei Darussalam, Gadong, Brunei Darussalam; 3 Estación de Biodiversidad Tiputini, Colegio de Ciencias Biológicas y Ambientales, Universidad San Francisco de Quito (USFQ), Quito, Ecuador

**Keywords:** *Corethrella*, Host specificity, Parasites, Phylogeny, Prevalence, *Trypanosoma*

## Abstract

**Background:**

Female frog-biting midges (*Corethrella*) are hematophagous micropredators that feed on frogs and serve as vectors for trypanosomes (*Trypanosoma*), unicellular flagellate parasites. Little is known about the infection ecology and host specialization within this tritrophic interaction.

**Methods:**

In this study, we explore the prevalence, diversity and specificity of *Trypanosoma* infections in *Corethrella* across various localities in tropical America and Borneo by sequencing both midge and trypanosome markers in midge samples.

**Results:**

Bayesian phylogenetic analyses and ASAP species delimitation of *Corethrella* (cytochrome c oxidase I [COI]) and *Trypanosoma* (18S, glyceraldehyde 3-phosphate dehydrogenase [GAPDH]) revealed a previously unknown high diversity of frog-biting midge-associated trypanosomes. Across regions and localities, the infection prevalence in midges caught by acoustic midge traps ranged from 2.9% to 23.5%, suggesting that a notable proportion of midges carried trypanosomes, likely acquired from a previous blood meal. At one locality, La Gamba (Costa Rica), the infection prevalence in trap-caught midges was 10.9%, while it was even higher in midges collected directly from frog hosts (20.7%), in agreement with the hypothesis that midges ingest trypanosomes from infected frogs. Bipartite network analyses revealed high degrees of specialization of *Trypanosoma* in trap-caught *Corethrella,* both across all localities (H2′ = 0.87) and when analyzed for our most sampled locality (Cahuita, Costa Rica) alone (H2′ = 0.94).

**Conclusions:**

Our data suggest that most trypanosomes detected in trap-caught midges are established, host-competent (i.e., specialist) parasites in an infective stadium.

**Graphical Abstract:**

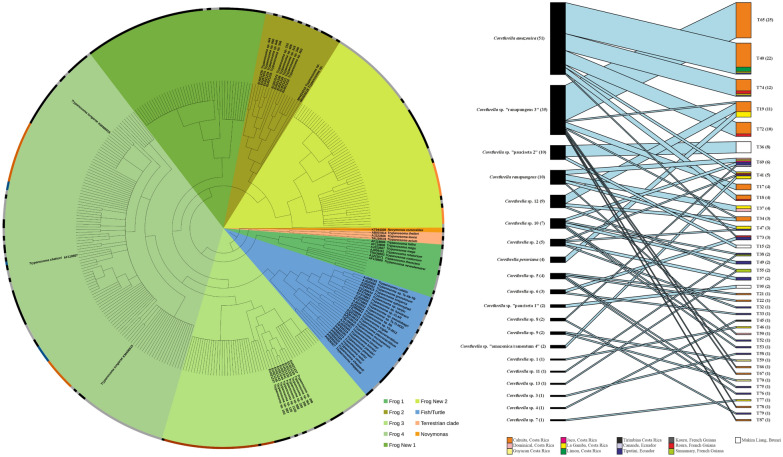

**Supplementary Information:**

The online version contains supplementary material available at 10.1186/s13071-025-06993-8.

## Background

Frog-biting midges (Diptera: Corethrellidae, with the monotypic genus *Corethrella*) are a family of blood-sucking midges whose females are specialized to feed on frogs, which they locate and approach on the basis of their mating calls [1]. The costs imposed by midges on frog hosts include irritations, indicated by defensive behaviors [2], and a potentially substantial blood loss [3]. Presumably, *Corethrella* also act as vectors for a potentially great variety of pathogens among amphibians. Here, we focus on the poorly studied association with *Trypanosoma* (Kinetoplastida: Trypanosomatidae), unicellular parasitic flagellate protozoa that infect a variety of invertebrate and vertebrate hosts and cause various diseases. 

More than 500 species of *Trypanosoma* are currently described [4]. However, the greater majority of Trypanosomatidae likely has not yet been discovered (e.g., [5]). While most genera within this family are monoxenous (i.e., single-host) parasites of invertebrates, *Trypanosoma* spp. have evolved a dixenous (i.e., host-switching) life cycle involving an invertebrate and a vertebrate host [6, 7]. The life cycles of digenic trypanosomes can be complex and differ substantially depending on the host species involved [8].

*Trypanosoma* spp. parasitize all classes of vertebrates and are distributed across all continents [8]. Most studies involve human-pathogenic species, especially *Trypanosoma cruzi* and *T. brucei*, the causative agents of Chagas Disease and African Sleeping Sickness (see in Vogel et al. [2021] [9] for a review). Despite their high abundance [10, 11] and increasingly acknowledged ecological relevance [12], there are few studies on non-human pathogenic trypanosomes. In general, *Trypanosoma* can be divided into a terrestrial and an aquatic clade [13], which share a monophyletic origin [8, 14]. While the terrestrial clade is mainly associated with mammals, birds, and terrestrial reptiles, the aquatic clade comprises species infecting amphibians, fish, and sea turtles [14]. Due to their biphasic life cycle, amphibians were suggested to represent a link between these two clades [15]. However, phylogenetic history in *Trypanosoma* is still not fully resolved (reviewed in Hamilton and Stevens [2017] [16]). Spodareva et al. [4] proposed that an ancestral leech-transmitted anuran trypanosome subsequently adapted to other vertebrate host taxa, including fishes and amniotes. To date, approximately 60 species of *Trypanosoma* are known to parasitize frogs [4], but it can be assumed that there is a much greater diversity. The dynamics of trypanosome infections in frogs and many aspects regarding their life cycles and host associations remain unknown.

High levels of polymorphism during the different life stages [17, 18], as well as mixed infections with multiple trypanosome species [4, 19, 20], make species identification at the morphological level difficult. Therefore, more recent studies increasingly rely on molecular genetic methods for the determination of trypanosome prevalence and diversity [17, 21]. Mixed infections of different *Trypanosoma* spp. in frogs [4] indicate that frogs act as intermediate hosts, functioning as reservoirs for various trypanosome species. This supports the general understanding that vertebrate hosts are more universal, whereas the interaction with invertebrates can be considered more specific due to their more complex development within the invertebrate host [8]. Bardsley and Harmsen [15] suggested that blood-sucking leeches act as the main trypanosome vectors among European frog populations. However, it was already known that insects can transmit trypanosomes in frogs: *Trypanosoma bufophlebotomi* is transmitted by sandflies (*Phlebotomus*) to toads (*Bufo bufo*) [22]. Subsequently, acoustically oriented frog-biting midges (Corethrellidae) were suggested as important vectors in the neotropics on the basis of a strongly biased infection prevalence of *Trypanosoma* in male (calling) frogs compared with silent females [21, 23]. *Corethrella* spp. are now considered the most important vectors of *Trypanosoma* in subtropical and tropical frog communities: In male Carolina tree frogs (*D. cinereus*), a nocturnal peripheral parasitemia was observed, which appeared in synchrony with the peak activity of the syntopic *Corethrella wirthi* [23], and trypanosomes were identified in *C. wirthi* mid- and hindguts [23].

In this study, we aim to provide a comprehensive assessment of the prevalence, diversity, and specificity of *Trypanosoma* infections among *Corethrella* across various tropical localities in Costa Rica, Ecuador, French Guiana, and Brunei Darussalam. We used polymerase chain reaction (PCR) and Sanger sequencing to detect and identify *Trypanosoma* DNA in female frog-biting midges. These midges were collected in two ways: (1) by directly sampling of feeding midges from their frog hosts and (2) by capturing midges with sound traps. We used DNA barcoding to reconstruct phylogenies for both taxa and to assess the specialization of *Trypanosoma* species in relation to their invertebrate (*Corethrella*) and vertebrate (frog) hosts.

## Methods

### Sampling of frog-biting midges

**Fig. 1:**
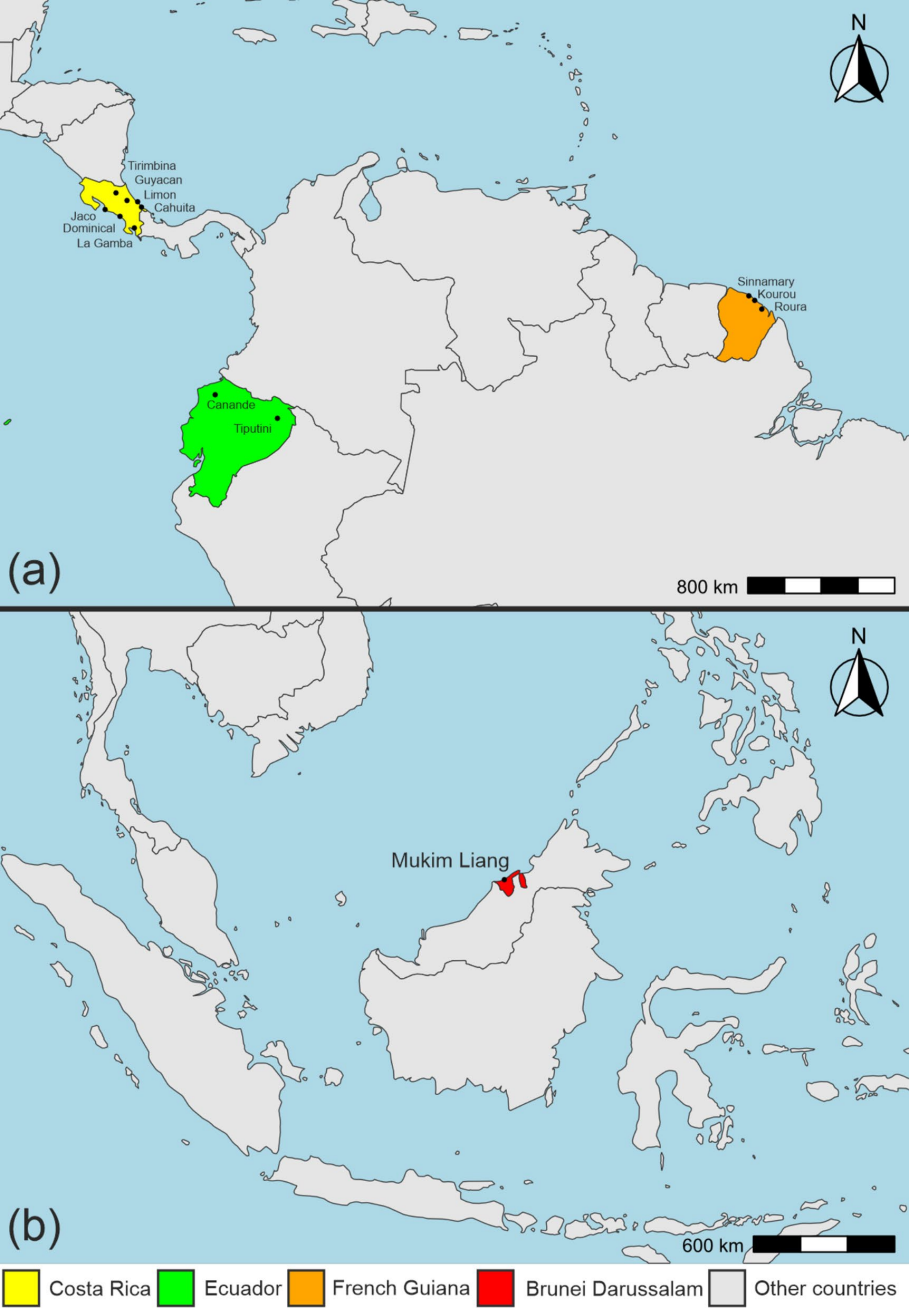
Sampling localities of *Corethrella* spp. **a** Sampling localities in Central and South America. *Corethrella* spp. were sampled in Costa Rica (Tirimbina, Guyacan, Limón, Cahuita, Jaco, Dominical, and La Gamba), Ecuador (Canande and Tiputini) and French Guiana (Sinnamary, Kourou, and Roura). **b** Sampling locality in Southeast Asia. *Corethrella* spp. were sampled in Brunei Darussalam (Mukim Liang)

Female frog-biting midges (*Corethrella* spp.) were collected at tropical lowland localities in Costa Rica, French Guiana, Ecuador, and Brunei Darussalam during 2013–2020 (Fig. 1). In Costa Rica, samples were collected at seven localities, including three on the Pacific coast (Jaco, Dominical, and La Gamba), two on the Atlantic coast (Limón and Cahuita), and two on the Atlantic slopes toward the central valley (Guyacan and Tirimbina). In French Guiana, midges were collected at three localities on the Atlantic coast (Sinnamary, Kourou, and Roura). Sampling localities in Ecuador included the Canande Reserve in the Chocó rainforest near the Pacific coast and the Tiputini Biodiversity Station located in the Amazon basin (Yasuní National Park) east of the Andes. In Brunei Darussalam, midges were collected in the Paya Gambut forest in Mukim Liang in the western part of the country.

Midges were collected with acoustic traps broadcasting a variety of recorded frog calls and synthetic sounds [24, 25]. At La Gamba, we also collected midges directly from frog hosts using aspirators during blood feeding. We grouped midges on the basis of morphological traits using a dissecting scope and assigned them to morphotypes or identified them to the species level when possible. For molecular genetic analyses, we chose a subset of these specimens, including as many *Corethrella* morphotypes/species and *Corethrella*-trap/frog interactions per site as possible.

Maps were created with R using publicly available data from Natural Earth (https://www.naturalearthdata.com/).

### DNA extraction, PCR, and sequencing

To investigate individual host–parasite interactions, genomic DNA was extracted separately from whole *Corethrella* specimens using the GeneReleaser (BioVentures Inc.) protocol adapted from Weigand [26] and established for *Corethrella* by Virgo et al. [27]. We performed DNA extractions and PCR for a total of 2645 midges, including 2079 individuals from traps and 566 individuals directly sampled from frog hosts (Table 2). For molecular identification of *Corethrella*, we used primers HCO2198/LCO1490 [28] to amplify an ~750 bp region of the mitochondrial cytochrome c oxidase I (COI) gene. *Trypanosoma* infection prevalence was determined via diagnostic PCR and subsequent Sanger sequencing, using primers TR-SSU1-F/TR-SSU1-R [21] to amplify an ~820 bp region of the *Trypanosoma* 18S ribosomal RNA (rRNA) gene (subunit 1 [SU1]) and primers G3 and G4a/G4b [14] to amplify an ~900 bp region of the glyceraldehyde 3-phosphate dehydrogenase (GAPDH) gene. PCR reactions of 12.5 µL were setup as follows for COI/18SSU1/GAPDH, respectively: 1/5/1 µL DNA template, 4.75/0.75/4,75 µL H_2_O, 6.25 µL GoTaq Colorless Master Mix (Promega), and 0.25 µL forward/reverse primers. The thermocycling protocols used for each marker are summarized in Table 1. All PCR products were purified using Exo1/FastAP (Thermo Scientific), and sequencing was performed on a capillary electrophoresis (CE) sequencer (Applied Biosystems 3130xl Genetic Analyzer) at Ruhr-University Bochum, Department of Receptor Biochemistry.

**Table 1:** Thermocycling protocols used for amplification of COI (*Corethrella* spp.), 18SSU1, and GAPDH (*Trypanosoma* spp.)

Marker	Initial denaturation	Cycles	Denaturation	Annealing	Extension	Final extension
COI	94 °C—3 min	40	94 °C—20 s	50 °C—20 s	72 °C—40 s	72 °C—5 min
18SSU1	94 °C—3 min	35	94 °C—30 s	54 °C—30 s	72 °C—60 s	72 °C—10 min
GAPDH (G3 & G4a)	94 °C—3 min	10/30	94 °C—30 s	62 °C—90 s/57 °C–90 s	72 °C—60 s	72 °C—5 min
GAPDH (G3 & G4b)	94 °C—3 min	10/30	94 °C—30 s	56 °C—90 s/51 °C—90 s	72 °C—60 s	72 °C—5 min

### Phylogenetic reconstruction

Editing and processing of nucleotide sequences were conducted using GeneiousPrime^®^ software (version 2019.2.1). Forward and reverse sequences were trimmed according to quality, with a cutoff value of > 5% error probability. Sanger-specific low-quality regions at the 5′ and 3′ ends were also removed. Sequences were aligned using the MAFFT plugin [29]. All alignments were visually inspected, with manual correction of sequencing errors, gaps, and inserts. Phylogenetic trees for both *Corethrella* (COI) and *Trypanosoma* (18SSU1/GAPDH) were built using Bayesian analyses implemented in Geneious (Mr Bayes version 3.2.6) [30]. Following Abadi et al. [31], we skipped a priori model selection and instead chose the most parameter-rich model GTR + I + G (four gamma categories) as a substitution model. Four MCMC chains (three hot/one cold) were run in a duplicate for 10,000,000 generations with a subsampling frequency of 5000 generations, using default temperatures and default prior distributions with unconstrained branch lengths. The first 2,500,000 generations were discarded as burn-in, and a majority rule consensus tree was constructed. The convergence of run parameters was assessed by visual inspection of trace/density plots and effective sample size (ESS) estimates (ESS threshold > 200). Trees were visualized and annotated using TreeViewer [32]. *Trypanosoma* 18SSU1 phylogeny was built using the newly generated sequences from PCR-positive midges and previously published sequences of a variety of *Trypanosoma* 18SSU1 sequences obtained from Genbank, aiming to assess *Trypanosoma*-diversity and phylogenic structure among our samples. Reference sequences have been previously used in other studies to classify *Trypanosoma* species [4, 21, 33]. *Trypanosoma* phylogenies (18SSU1/GAPDH) were rooted using Basic Local Alignment Search Tool (BLAST)-Hit sequences of the insect trypanosomatid *Novymonas esmeraldas* (Genbank Accession MW694343/KT944308). The *Corethrella* COI tree was rooted using BLAST-Hit Genbank sequence of the mosquito species *Culex nigropunctatus* (Genbank Accession AB738113).

In addition to the tree-based (visual) species delimitation, we used the ASAP web tool ([34]; https://bioinfo.mnhn.fr/abi/public/asap/asapweb.html) to calculate a barcoding gap. We ran the web application using the Kimura-2 parameter distance model with default parameter settings. We selected the partition output (i.e., the number and composition of genetic clusters) that best matched the species-level resolution of the reference sequences obtained from GenBank. For *Corethrella*, we chose the output with the lowest ASAP score and best fitting threshold distance [34].

To further reconstruct the species delimitation of *Trypanosoma*, newly generated sequences available for both marker genes (18SSU1/GAPDH) were visualized in a tanglegram using the *phytools* package in R [35]. To quantify the consensus of both phylogenies, we used the Procrustean Approach to Cophylogeny (*PACo*) method with the R package *PACo* [36, 37] choosing 100,000 random permutations.

### Shannon–Wiener index and evenness

To evaluate trypanosome diversity across sampling sites, we calculated the Shannon–Wiener diversity index (*H*′) and Pielou’s evenness (*J*′) on the basis of the distribution of trypanosome 18S molecular operational taxonomic units (MOTUs) as delimited by ASAP (phylogenetic reconstruction). Indices were only calculated for localities with more than ten total trypanosome detections. Both indices were computed in R using the *vegan* package [38].

### Bipartite interaction network

To assess host specialization, we used the *bipartite* package [39] in R, based on the COI and 18SSU1 phylogenies and MOTUs provided by ASAP, with trap-caught midges and their associated trypanosomes across all countries and for Cahuita, Costa Rica, respectively. Network structure was analyzed using the following metric, as described by Dormann et al. [39]: the quantitative weighted specialization index H2′, which estimates the overall specificity of the network ranging from 0 (indicating no specificity) to 1 (indicating maximum specificity). To evaluate deviations from randomly structured networks, the observed H2′ values were compared with those from null models that maintained marginal totals while randomly shuffling interactions (10,000 permutations, *t*-test).

## Results

Among trap-catches, *Trypanosoma*-infection prevalence ranged from 2.9% to 23.5% for the different localities (Table 2) with an overall mean of 9.0% (*σ* = 5.19; *N* = 211). The infection prevalence for directly sampled *Corethrella* (only at La Gamba, Costa Rica) was 20.7% (*N* = 117).

**Table 2:** Sampling localities and corresponding numbers of *Corethrella* spp. collected and *Trypanosoma* spp. detected (PCR, 18SSU1)

Country	Site	*Corethrella*		*Trypanosoma*			
		Sampling method	*N*	PCR pos.^a^	Prevalence (%)	18SSU1 sequence^b^	Shannon–Wiener index (evennes)
Costa Rica	La Gamba, Tropical Station La Gamba	Direct	566	117	20.7	85	2.388 (0.843)
		Acoustic	230	25	10.9	24	1.955 (0.849)
Costa Rica	Guyacan, CRARC Rainforest Reserve	Acoustic	58	3	5.2	3	—
Costa Rica	Limón	Acoustic	43	4	9.3	4	—
Costa Rica	Dominical	Acoustic	64	6	9.4	6	—
Costa Rica	Jaco	Acoustic	52	4	7.7	3	—
Costa Rica	Cahuita	Acoustic	370	87	23.5	84	2.214 (0.798)
Costa Rica	Sarapiquí, Tirimbina Biological Reserve	Acoustic	54	2	3.7	1	—
Ecuador	Tiputini, Yasuní National Park	Acoustic	403	28	6.9	25	2.620 (0.945)
Ecuador	Esmeraldas, Canande Reserve	Acoustic	136	4	2.9	4	—
French Guiana	Sinnamary	Acoustic	69	10	14.5	9	—
French Guiana	Roura	Acoustic	201	12	6.0	10	2.025 (0.974)
French Guiana	Kourou	Acoustic	38	4	10.5	5	—
Brunei Darussalam	Mukim Liang	Acoustic	361	22	6.1	23	1.627 (0.782)
			2645 (total)	328 (total)	9.8 (mean)	286 (total)	

CRARC, Costa Rican Amphibian Research Center; pos., positive

^a^All positive PCR calls (18SSU1); includes co-amplification of other Trypanosomatidae DNA

^b^High-quality sequences obtained via Sanger sequencing

We obtained high-quality 18SSU1 sequences for 201 (95.3%) of the trap-based PCR-positive samples, whereas for direct samples the sequencing success was considerably lower (72.6%) with 85 high-quality sequences. Overall, sequence quality was diminished by overlapping signals of co-amplified Trypanosomatidae DNA, indicating co-infections with multiple *Trypanosoma* spp. (see discussion). Of the obtained high-quality sequences, 18 were unambiguously identified via BLAST searches in Genbank as *Novymonas esmeraldas*—a monoxenous trypanosomatid infecting a variety of insects [40]. *N. esmeraldas* was detected in samples from Costa Rica, including multiple *Corethrella* spp. caught in different years with traps (Cahuita) as well as directly from frogs (La Gamba). Additionally, *N. esmeraldas* was found in a single sample from Brunei and in two samples from Ecuador. *Novymonas* infections were not included in all further analyses.

To reconstruct phylogenies and analyze parasite host associations, 152 paired *Trypanosoma* 18SSU1 and *Corethrella* COI sequences were used. High-quality GAPDH sequences were obtained for only 86 of the 18SSU1 sequences (30.1%) due to insufficient amplification. Although the phylogenies of *Trypanosoma* 18SSU1 and GAPDH showed significant congruence (Fig. S2), as revealed by *PACo* (m^2^_observed_ = 0.018, *P* < 0.01), we limited our further analyses to 18S because of the larger sample size.

**Fig. 2:**
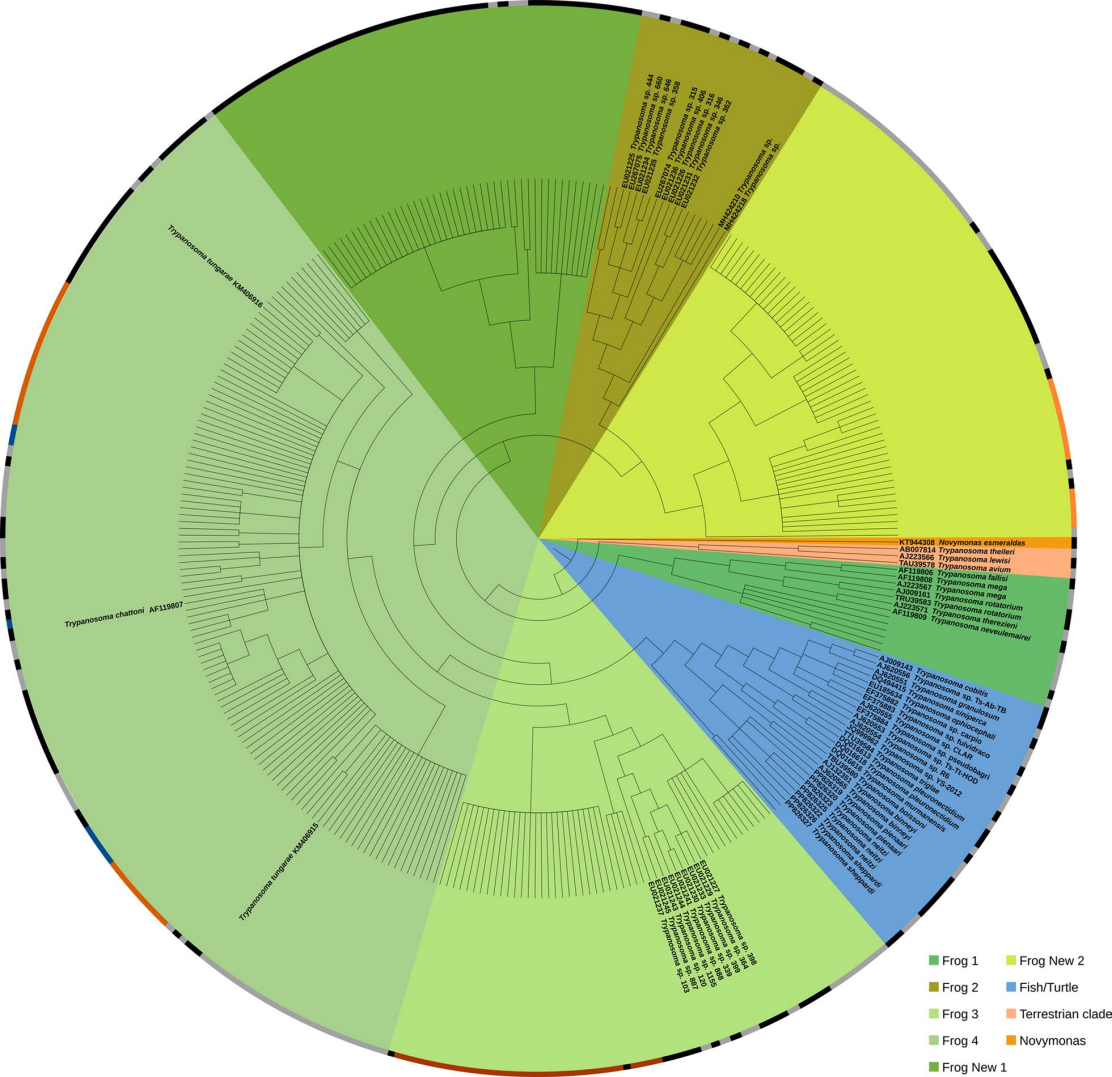
Cladogram of *Trypanosoma* spp. based on 18S rRNA Sanger sequencing data, isolated from frog-biting midges (*Corethrella* spp.). Unlabelled nodes were isolated from frog-biting midges (*Corethrella* spp.) collected in Costa Rica, Ecuador, French Guiana, and Brunei Darussalam (this study). Labelled nodes represent reference sequences obtained from GenBank (*N* = 53), including the GenBank Accession Number and species. Reference sequences of the clades Frog 1 to Frog 4 were isolated from frogs, and reference sequences of the clade Fish/Turtle consist of known species isolated from freshwater and saltwater fish and turtles. Frog New 1 and Frog New 2 consist exclusively of trypanosomes detected in this study, isolated from frog-biting midges. Borders indicate MOTUs as defined by the ASAP web tool. Colored segments highlight identical MOTUs that are discontinuous in the phylogenetic tree due to interruption by other MOTUs. In total, 102 MOTUs were identified. In total, 268 sequences were isolated from frog-biting midges, representing 59 MOTUs. For clarity, some tip labels were omitted from the figure. A fully labeled version is available online (https://figshare.com/s/38c88f5d3fafe9d142d9)

*Corethrella* COI barcoding revealed 25 haplotype clusters, supported by high Bayesian posterior probabilities (> 0.95) and a distinct barcoding gap, based on Kimura 2-parameters (K2P) distances (intraspecific: < 6.0%; interspecific: 11–35%). Some of those clusters could be referenced to known species [27] (Fig. 2). Six clusters were represented only by singleton midges, whereas the largest cluster (*C. amazonica*) contained 51 specimens. MOTU clustering in *Corethrella* COI was congruent for both delimitation methods used (Appendix, Fig. S1).

For the *Trypanosoma* 18S tree, MOTU delimitation was not as conclusive, likely due to an overall lower variability of this marker, with average overall sequence identities of 95.2%. Here, no clear barcoding gap was found, and ASAP detected 32–102 MOTUs. For the GenBank-referenced tree in Fig. 2, we selected the output that best matched the species-level resolution of the reference sequences obtained from Genbank, which also had the lowest ASAP score, resulting in 102 MOTUs overall. In that output the 53 different species from GenBank were partitioned into 43 MOTUs, indicating that our approach is conservative.

Most of our samples grouped with known frog-associated *Trypanosoma* spp. belonging to the aquatic clade. One cluster (PQ889788, PQ889789) isolated from midges in Brunei was associated with an aquatic subclade previously found in marine and freshwater fishes and turtles. Among the frog-associated clades, a large proportion of our samples grouped together with *Trypanosoma tungarae* (KM406915, KM406916 [21]). Our findings suggest that *T. tungarae* isolates KM406915/KM406916, previously described by Bernal and Pinto [21], may harbor multiple *Trypanosoma* species.

A single sample clustered together with two samples associated with sand flies (Psychodidae) in Brazil (EU021234, EU021235; [41]), while all other samples (59 MOTUs) likely represent so far undescribed *Trypanosoma* spp. Additionally, we identified two novel clades, consisting of 6 and 12 MOTUs, respectively, with high Bayesian posterior probabilities (Frog(midge) New 1, Frog(midge) New 2; Fig. 2).

**Fig. 3:**
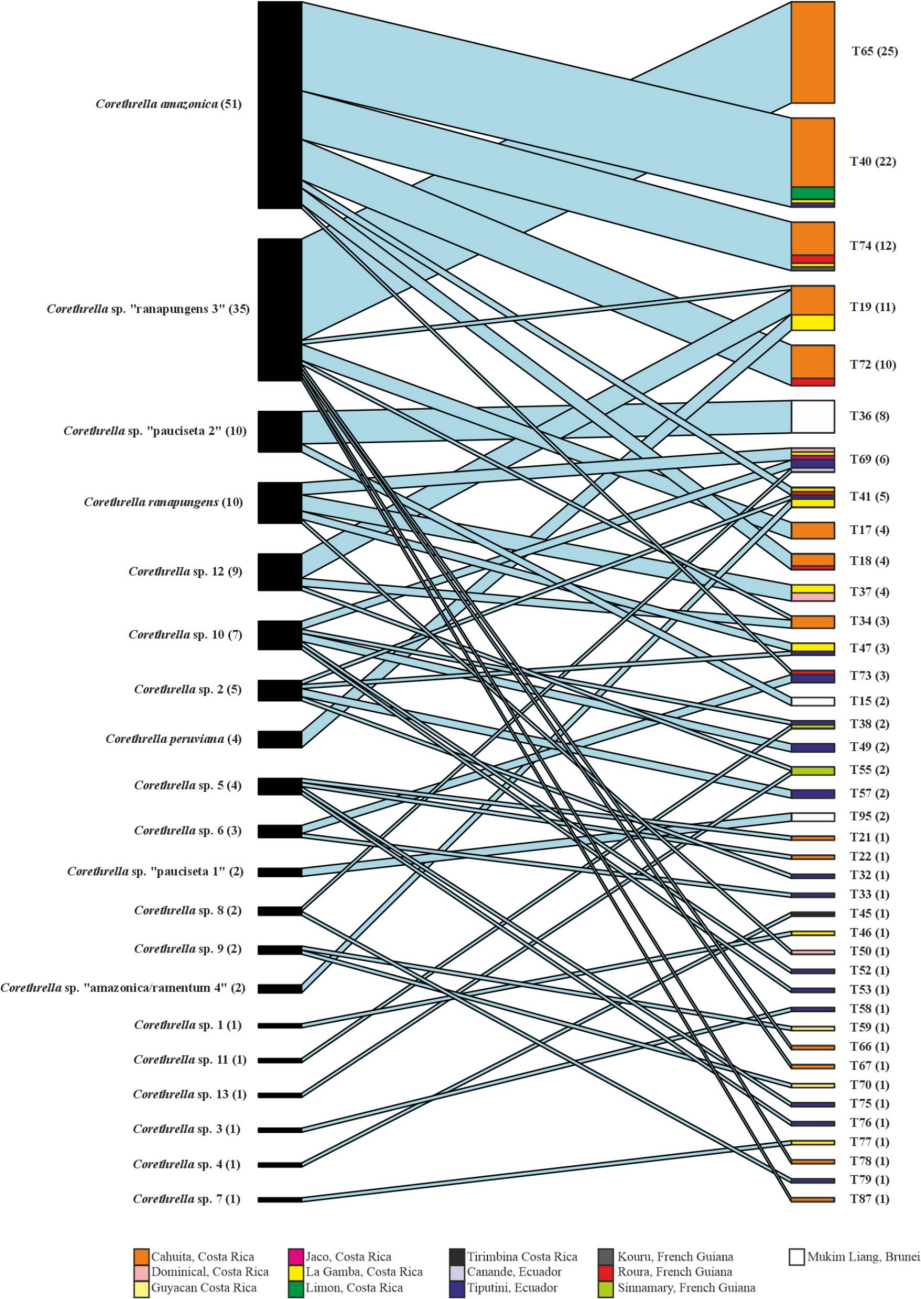
Quantitative bipartite interaction network showing high levels of specialization (H2′ = 0.87) among trap-caught frog-biting midges (*Corethrella* spp.) and associated trypanosomes (*Trypanosoma* spp.) from Costa Rica, Ecuador, French Guiana, and Brunei Darussalam. MOTUs were inferred on the basis of Sanger sequencing data of COI (*Corethrella*) and 18SSU1 (*Trypanosoma*), and via species delimitation algorithms provided in the ASAP online tool [34]. Values in parenthesis indicate sample size. MOTUs are arranged in descending order based on their total number of interactions in the network

**Fig. 4:**
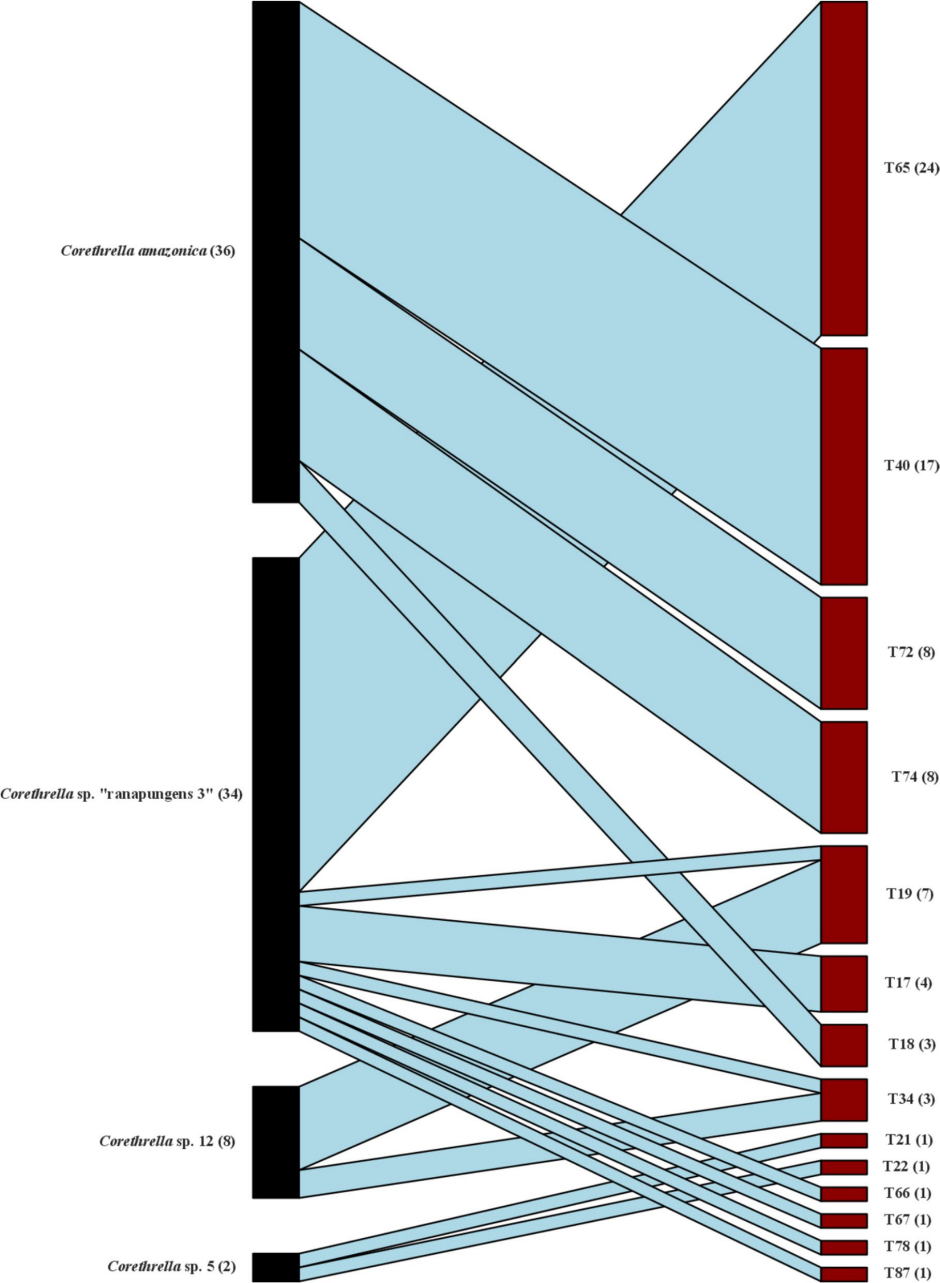
Quantitative bipartite interaction network showing high levels of specialization (H2′ = 0.94) among trap-caught frog-biting midges (*Corethrella* spp.) and associated trypanosomes (*Trypanosoma* spp.) from Cahuita, Costa Rica. MOTUs were inferred on the basis of Sanger sequencing data of COI (*Corethrella*) and 18SSU1 (*Trypanosoma*), and via species delimitation algorithms provided in the ASAP online tool [34]. Values in parenthesis indicate sample size. MOTUs are arranged in descending order based on their total number of interactions in the network

Bipartite network analysis indicated high degrees of specialization (Figs. 3, 4). Across all localities and regions, the network-level specialization H2′ was 0.87 with individual degrees of specialization ranging from generalist to specialist interactions. The network showed many specialized links, mainly on the parasite side (Fig. 3). A large portion of the *Trypanosoma* MOTUs were derived from only a few or single midge individuals, such as T15 or T21. The three MOTUs detected in the largest numbers of midge samples, T65 (*N* = 25), T40 (*N* = 22), and T74 (*N* = 12), were found exclusively in a single midge species (*Corethrella* sp. “ranapungens 3” [T65]) and *Corethrella amazonica* (T40 and T74). Among those, the MOTUs T40 and T74 were composed of individuals from more than four distinct localities, demonstrating *Trypanosoma* specialization across localities in Central America as well as across the Neotropics (Fig. 3). In general, multiple *Trypanosoma* MOTUs with varying degrees of specialization are distributed across Central and South America. High levels of specialization were also found in both midge and *Trypanosoma* MOTUs from Brunei; however, they represented distinct, geographically restricted lineages (T36 and T95; *Corethrella* sp. “pauciseta 1”, *Corethrella* sp. “pauciseta 2”). Most *Corethrella* MOTUs carried a variety of *Trypanosoma* MOTUs, except for those that were represented by only single or a few midge individuals, such as *Corethrella peruviana* or *Corethrella* sp. 3.

For Cahuita, bipartite network analysis revealed an even higher H2′ value of 0.94. Except for two cases (T19, T34), all MOTUs were found exclusively in association with a single midge MOTU (Fig. 4).

The observed specializations were significantly higher than expected under the null models (all: *t*-test, *t*_(9999)_ = −2481.1, *P*-value < 0.0001; Cahuita: *t*-test, *t*_(9999)_ = −2024.9, *P*-value < 0.0001).

## Discussion

In our study, we used molecular markers to delineate putative species and depict the interaction web of *Trypanosoma* and *Corethrella* to explore host associations. Our analysis revealed a previously unrecognized diversity among *Corethrella*-associated *Trypanosoma* species, including the discovery of two new clades within the large aquatic clade. The inclusion of GenBank sequences enabled a phylogenetic reconstruction of the aquatic clade that was consistent with earlier findings [4, 21, 33]. Although our results should be considered preliminary with regard to basal phylogeny due to known limitations of the 18S gene [14], the strong congruence with our GAPDH data supports the reliability of our 18S-based species delineation.

*Trypanosoma*-positive midges were detected in acoustic trap samples at all investigated localities, albeit in varying proportions. If *Trypanosoma* infections in *Corethrella* occur exclusively through the ingestion of infected frog blood, our data suggest that, on average, at least 9.0% of trap-caught midges have previously fed on *Trypanosoma*-infected blood. However, it remains unclear what proportion of these positives was derived from *Trypanosoma*-carrying blood meals yet residing in the digestive tract, and what proportion represented host-competent *Trypanosoma* already established in the midge. At present we can only indirectly address this question by comparing rates of positives between trap-caught midges that had not fed at least for hours (or, presumably more often, not at all) and those sampled directly with an aspirator when feeding on frog hosts. In La Gamba, the latter had about twice as many positives as trap-caught midges, suggesting that these additional positives stemmed from the recent blood meal. Direct sampling also resulted in a lower quality of *Trypanosoma* Sanger sequences, many of which showed signs of sequence overlap. This is in agreement with the finding that a frog may host more than one *Trypanosoma* species [23]. After blood uptake, only those *Trypanosoma* species that are compatible with the host midge would survive and complete their life cycle, resulting in overall more specialized *Trypanosoma*–midge associations. The high degrees of *Trypanosoma*–*Corethrella* specialization observed in our bipartite network analyses is in agreement with this view, suggesting that most trypanosomes detected in trap-caught midges are established, host-competent parasites in an infective stadium.

Several studies support the assumption that *Trypanosoma* generally exhibit lower specialization to their vertebrate hosts than to their invertebrate host. Sehgal and Smith [42] documented a widespread mix of trypanosomes across various African bird taxa and geographical locations. *Trypanosoma vivax*, the causative agent of African Trypanosomiasis in livestock, has an extensive host range, including nine domestic mammals and nearly 40 wild fauna species [43]. Similarly, Ray and Choudhury [44] reported the same *Trypanosoma* species occurring across a variety of Indian frog species. However, few studies have investigated host specificity in the invertebrate host. A prominent example is *T. vivax*, which is primarily transmitted by Tsetse flies (*Glossina* spp., Glossinidae). Here, the parasite undergoes sexual reproduction and reaches an infective stage in the invertebrate (fly) host (see Osório et al. [2008] [45] for a review). While *T. vivax* can also be transmitted by distantly related hematophagous flies such as *Tabanus* spp. (Tabanidae), *Stomoxys calcitrans,*, and *Haematobia irritans* (both Muscidae) (see Fetene et al. [2021] [43] for a review), the vector competence of these alternative hosts has not been thoroughly explored. Finally, Oberle et al. [46] demonstrated that, during the life cycle of different *Trypanosoma brucei* strains, strong population bottlenecks occur during the parasites’ development within the foregut of the invertebrate vector. Over time, this could have led to specialization on particular invertebrate vectors, as only specific parasite lineages are able to successfully complete their life cycle.

Our bipartite network analysis is consistent with a high degree of specialization on the invertebrate side, as *Trypanosoma* species do not associate randomly with *Corethrella* hosts. Midges captured with sound traps revealed many specialized links mainly on the parasite side. These specialized interactions were sometimes associated with locality, as several *Trypanosoma* MOTUs, including some frequently encountered ones (e.g., T65), were only recorded from single localities. Not surprisingly, the local interaction network for Cahuita, our best sampled single locality, revealed a very high degree of specialization. Here, 12 of 14 *Trypanosoma* MOTUs were detected exclusively in a single midge MOTU (Fig. 3). Interestingly, some of the observed specializations were maintained across Central American localities and, in two cases, even across regions, i.e., between Central America and the western Amazon (T40) or between Central America and the Guiana Shield (T74). Although these transregional cases of specialization are based on only a few detections from South America (*N* = 1–2), they underline a pattern of substantial host specificity. It is noteworthy that they were both found in association with the same single species of frog-biting midge, *Corethrella amazonica*, the midge species with the highest number of detected infections in this study. This matches previous studies, which describe *C. amazonica* as one of the most widespread frog-biting midge species, ranging from the Yucatán Peninsula in Mexico southward to Colombia, Trinidad and Tobago, Guyana, French Guiana, and Brazil [47, 48]. According to Poulin et al. [49], host specificity is indicated through the stable use of hosts despite variations in the host landscape. Although our data suggest consistency in host use across different localities, these results must be interpreted with caution, as our data clearly represent only a fragmentary snapshot of the true interaction network. More representative sampling at the included localities might broaden the apparent interaction specificity, as specialized *Trypanosoma* MOTUs are detected in additional midge species. However, it could also corroborate strong specialization in rarer MOTUs that have so far been only insufficiently sampled. We believe that our results are robust enough to give a first approximation of *Corethrella*–*Trypanosoma* interaction specificity, at least in the neotropics. Aside from additional sampling, the use of next-generation sequencing is desirable to improve *Trypanosoma* identification in multiply-infected hosts. Furthermore, research on *Trypanosoma* specialization would clearly benefit from better knowledge regarding infection and transmission pathways.

## Conclusions

Our results revealed a previously unrecognized diversity and specialization of *Trypanosoma* spp. associated with frog-biting midges (*Corethrella*). The consistent detection of infections across regions and the higher prevalence in midges collected directly from frogs support the hypothesis that midges acquire *Trypanosoma* through blood meals from infected frog hosts. The high degrees of *Trypanosoma*–*Corethrella* specialization observed in bipartite network analyses suggest that most trypanosomes detected in trap-caught midges are established, host-competent parasites in an infective stadium.

## Supplementary Information

Additional file 1 (Fig. S1: Dendogram of *Corethrella* spp. based on COI sequencing data. Midges were collected directly from their hosts and with acoustic traps at various locations in Costa Rica, Ecuador, French Guiana, and Brunei Darussalam. Species delimitation was performed using the ASAP web tool.

## Data Availability

Data are deposited at Figshare and will publicly available as of the date of publication (https://figshare.com/s/38c88f5d3fafe9d142d9).
