# Supplementary figures and images for: Uninvited guests: diversity and specificity of Trypanosoma infections in frog-biting midges (Corethrella spp.)
Source: Parasit Vectors. 2025 Aug 15;18:348. doi: 10.1186/s13071-025-06993-8 (PMC12355796; doi:10.1186/s13071-025-06993-8)

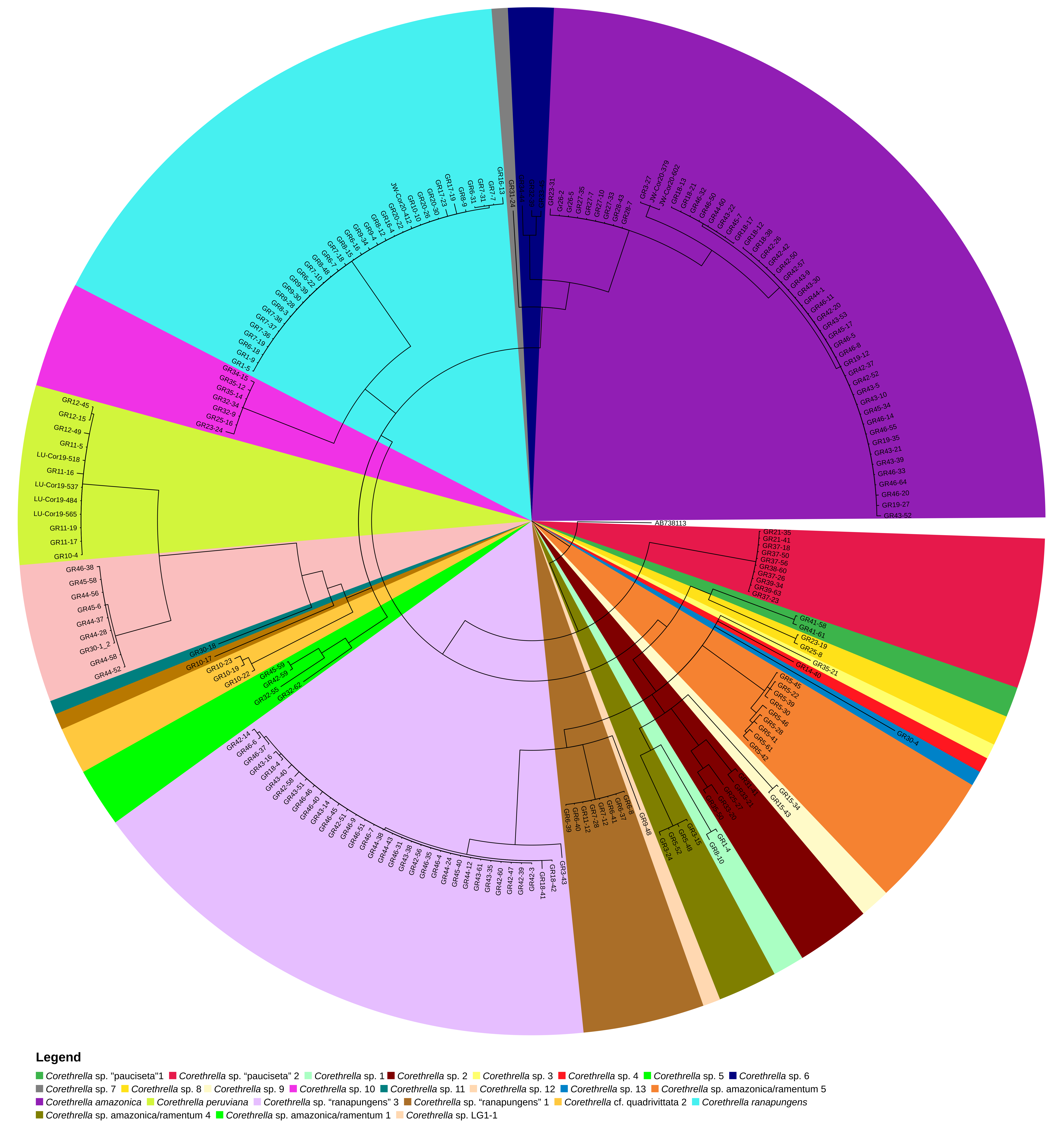

Supplement: Supplementary file 1 — Additional file 1 (Fig. S1: Dendogram of Corethrella spp. based on COI sequencing data. Midges were collected directly from their hosts and with acoustic traps at various locations in Costa Rica, Ecuador, French Guiana, and Brunei Darussalam. Species delimitation was performed using the ASAP web tool. [file 13071_2025_6993_MOESM1_ESM.png]
